# The Novel Use of PVP K30 as Templating Agent in Production of Porous Lactose

**DOI:** 10.3390/pharmaceutics13060814

**Published:** 2021-05-30

**Authors:** Wei-Feng Zhu, Lin Zhu, Zhe Li, Wen-Ting Wu, Yong-Mei Guan, Li-Hua Chen, Zhi-Xuan Mao, Liang-Shan Ming

**Affiliations:** Key Laboratory of Preparation of Modern TCM, Ministry of Education, Research Center for Differentiation and Development of TCM Basic Theory, Institute for Advanced Study, Jiangxi University of Chinese Medicine, Nanchang 330004, China; zwf0322@126.com (W.-F.Z.); 201981800034@jxutcm.edu.cn (L.Z.); 20131052@jxutcm.edu.cn (W.-T.W.); 20181015@jxutcm.edu.cn (Y.-M.G.); 19940081@jxutcm.edu.cn (L.-H.C.); 201801006044@jxutcm.edu.cn (Z.-X.M.)

**Keywords:** lactose, PVP K30, templating agents, porous, dissolution behavior

## Abstract

It is necessary to prepare porous lactose in order to improve the dissolution behavior of insoluble active ingredient. In this study, polyvinylpyrrolidone K30 (PVP K30) was firstly utilized as a templating agent with different use levels in preparing porous lactose. Then, the physical properties were profoundly characterized. Finally, the porous lactose was also employed as a health functional food/drug carrier to explore the effect on the dissolution behavior of curcumin. The results confirmed that (i) porous lactose was successfully prepared using PVP K30 as templating agent; (ii) PVP K30 significantly improved the yield of lactose in the spray drying; (iii) the improved powder properties of porous lactose were more conducive to the downstream operating process for the preparation of health functional food or drug; and (iv) the porous lactose significantly improved the dissolution behavior of curcumin. Therefore, the results obtained are beneficial to boosting the development of porous materials.

## 1. Introduction

Natural medicine and health functional foods, commonly originating from natural plants, have developed steadily over the years and have a long history of clinical practice [1,2]. The most common and classic health functional foods in China are also from natural plants [3], e.g., ginger, codonopsis pilosula, etc. Nowadays, natural medicine and health functional food receive an increasing acceptance because of their vital role in disease prevention [4,5,6]. Moreover, the occurrence of an aging society and the current health needs of people have further led to the improvement of the natural medicine and health functional food market and the promotion of the development of the natural medicine and health functional food industry [7,8].

Lactose is the most commonly used food and/or pharmaceutical excipient with its cost effectiveness, excellent physical/chemical stability, water solubility, light flavor, and low sweetness, caloric value, and glycemic index [9,10]. However, the major drawback in using lactose as a carrier for health functional food/drug loading purposes is its poor porosity and loading, as it has a low specific surface area [11] and thus a relatively poor dissolution behavior [12]. However, dissolution behavior is an important property affecting therapy efficacy of health functional foods and drugs [13,14]. Therefore, porous lactose is needed to be prepared in order to increase the loading capacity and improve the dissolution behavior of insoluble active ingredients [15,16].

Over the past decades, porous materials have been widely used in many applications where their unique properties include high surface-to-volume ratios [17,18]. Nowadays, co-spray drying assisting a templating agent is the most popular method for the production of porous material [11,19,20]. Spray drying is a typical technique to fabricate non-toxic microparticles using sugars in food and pharmaceutical particle engineering. In fact, the preparation process of the porous lactose contains two steps, i.e., spray drying and ethanol washing. Spray drying is mainly to prepare composite particles containing templating agent, while ethanol washing is mainly to remove the templating agent [19].

There are two key points in the process: (i) both lactose and the templating agent are soluble in water; (ii) lactose is insoluble in ethanol, while the templating agent is soluble in ethanol. Nowadays, the most commonly templating agents mainly focus on sucrose, citric acid, D-maltose monohydrate, D-fructose, D-glucose anhydrous, etc. [11,19,20,21,22], but attention is rarely paid to polyvinylpyrrolidone (PVP), which is soluble in both water and ethanol. Moreover, PVP can prevent the wall sticking during the spray drying process and therefore improve the yield.

In light of the above, this study aimed to (i) study the possibility of using PVP K30 as a templating agent, (ii) develop porous lactose, and (iii) explore the effect of porous lactose on the dissolution behavior of the insoluble active ingredient curcumin, which was extracted from ginger.

## 2. Materials and Methods

### 2.1. Materials

PVP K30 (Ashland, Wilmington, DE, USA), lactose (MEGGLE Excipients & Technology Group, Wasserburg am inn, Germany), and curcumin (Xi’an Hao-Xuan Bio-Tech Co., Ltd., Xi’an, China).

### 2.2. Preparation of Materials 

#### 2.2.1. Porous Lactose

PVP K30 was chosen as the templating agent. The samples were carried out by varying the templating agent (1%, 2%, 3%) (*w*/*w*) of the aqueous solution, with the lactose concentration kept constant at 10% (*w*/*w*). All solutions were magnetically stirred at a room temperature of 25 °C for at least 30 min, so a clear solution was obtained without any visible particles being present. The clear solutions were then spray-dried. The freshly spray-dried powder was collected, and some of the powder was mixed with enough ethanol, and the suspension was magnetically stirred for 24 h at the room temperature of 25 °C to remove PVP K30. Then porous lactose was separated from ethanol containing PVP K30 by centrifugal force, and dried in a vacuum chamber (−0.1 MPa) at 45 °C for 6 h.

Yield (%) was calculated with the following equation:

(1) $$Yield=\frac{m_{1}}{m_{0}}$$

where *m*_1_ (g) was the actual mass of freshly spray-dried powder, *m*_0_ (g) was the mass of the raw lactose for Lactose-0 and the mass of raw lactose and PVP K30 for Lactose-P1, Lactose-P2, and Lactose-P3.

#### 2.2.2. Raw Lactose and Non-Porous Lactose

The raw lactose was also characterized in this study. The non-porous lactose (Lactose-0) was processed without PVP K30, and the process was same with the porous lactose (Section 2.2.1).

### 2.3. Physical Characterizations

#### 2.3.1. Surface Morphology

The morphology of materials was examined under scanning electron microscope (SEM) (Quanta FEG 250, Philips Ltd., Eindhoven, The Netherlands) at an acceleration voltage of 20 kV. Samples were sputter coated (Leica EM ACE600, Leica Biosystems, Vienna, Austria) with gold-palladium and observed at different magnifications.

#### 2.3.2. Surface Areas, Pore Volumes, and Pore Diameter

Surface areas (SA), pore volumes (PV), and pore diameter (PD) of samples were characterized by surface area and pore volume analyzer (TriStar3000, Micromeritics Instrument Corp., Norcross, GA, USA). Nitrogen adsorption isotherms of samples were recorded at the temperature of liquid N_2_ (77 K). Consequently, SA was calculated from the Brunauer–Emmet–Teller (BET) equations and Barrett–Joyner–Halenda (BJH) method, respectively. PV and PD were calculated from BJH method.

#### 2.3.3. Particle Size (d0.5) and Size Distribution (Span), and Uniformity (Un)

The d (0.5), span, and Un values were determined by laser diffraction (Malvern 2000, Malvern Instruments Ltd., Malvern, UK; dry method). Each sample was tested three times.

#### 2.3.4. Carr’s Index (CI) and Hausner Ratio (HR)

The bulk density (*ρ_b_*) and tapped density (*ρ_t_*) were determined by a powder characteristics tester (BT-1000, Bettersize Instruments, Ltd., Shanghai, China). The measuring cylinder was exactly 100 mL. The tapped time was 6 min for each material. The CI and HR were calculated with the following equations:

(2) $$CI=\frac{\rho_{t}-\rho_{b}}{\rho_{t}}\times 100$$

(3) $$HR=\frac{\rho_{t}}{\rho_{b}}$$

#### 2.3.5. X-ray Diffraction (XRD)

XRD structural patterns of materials were characterized by an X-ray diffractometer (D8 Advance, A25, Bruker Ltd., Berlin, Germany). The crystal form of Lactose, Lactose-0, Lactose-P1, Lactose-P2 and Lactose-P3 was detected and analyzed by an X-ray diffractometer with Cu Kα1 radiation at 30 mA and 40 kV, and the samples were scanned from 3 to 80° at a scanning rate of 1 step/s with a 0.02° step size.

#### 2.3.6. Fourier Transform Infrared Spectrometer (FTIR) 

FTIR Spectrum Two (PerkinElmer, Liantrisant, UK) was used to investigate the components of lactose. Samples were prepared by mixing with potassium bromide (KBr) at a ratio of 1:10 and then compressed with hydraulic press at a pressure of 7 tones. The samples were scanned against a blank KBr disk ranging from 4000 to 400 cm^−1^ with a resolution of 1.0 cm^−1^.

#### 2.3.7. Thermal Gravity (TG) and Differential Thermal Gravity (DTG) Analysis

The thermal kinetic studies of samples were performed using an Exstar TG/DTA6300 TG analyzer (SII Nano, Japan). For this purpose, about 8 mg of the samples were taken in the pre-balanced ceramic pan and heated from 30 to 800 °C at heating rates of 10 °C/min under a flow of nitrogen of 200 mL/min.

### 2.4. Drug Loading and In Vitro Dissolution Behavior

#### 2.4.1. Drug Loading

100.0 mg curcumin was dissolved in 30.0 mL ethanol to obtain the drug solution, which was then mixed with 5.0000 g porous lactose for 4 h with a magnetic stirrer. After centrifugation, the curcumin-loaded lactose was pre-dried by inert nitrogen gas and oven-dried at 60 °C to a constant weight.

#### 2.4.2. In Vitro Dissolution Behavior

100.0 mg curcumin-loaded lactose was put into capsule to explore the dissolution behavior. The dissolution behavior was characterized with intellective dissolution analyzer (ZRS-8G, TIANDA TIANFA pharmaceutical testing instruments, Ltd., Tianjin, China). The basket-stirring method was employed. Aqueous solution with 5% Tween 80 (900 mL) was used as the dissolution medium. The temperature was 37 ± 0.5 °C. The speed was 100 rpm. Samples (2.5 mL) were taken and replaced with fresh dissolution medium immediately at 5, 10, 15, 30, 45, 60, and 90 min. The samples were filtered through a 0.45 mm filter and analyzed at 424.5 nm using a UV-vis spectrometer (UV-2600, Shimadzu Co., Kyoto, Japan).

## 3. Results and Discussion

### 3.1. Yield and Surface Morphology of Materials

The yield of porous lactose was shown in Table 1. Compared with the non-porous lactose processed without PVP K30 (Lactose-0), the yield of porous lactose processed with 1% PVP K30 (Lactose-P1), 2% PVP K30 (Lactose-P2), and 3% PVP K30 (Lactose-P3) increased 23.6%, 24.0%, and 29.1%, respectively. This demonstrated that PVP K30 could significantly prevent the wall sticking during the spray drying process. It may be due to the PVP K30 forming a thin coat over lactose.

**Figure 1:**
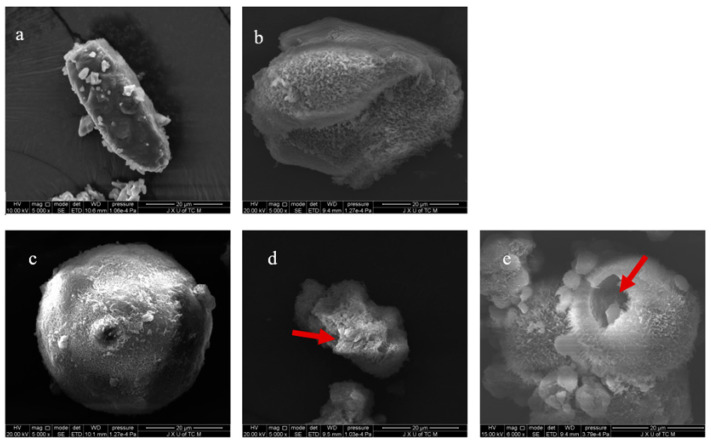
The scanning electron photomicrographs of materials. (**a**), Lactose (the raw material) (5000 X); (**b**), Lactose-0 (processed without PVP K30) (5000 X); (**c**), Lactose-P1 (processed with 1% PVP K30) (5000 X); (**d**), Lactose-P2 (processed with 2% PVP K30) (5000 X); (**e**), Lactose-P3 (processed with 3% PVP K30) (6000 X).

The surface morphology of particles was summarized in Figure 1, which apparently demonstrated that porous lactose was prepared successfully through spray drying with employing PVP K30 as templating agent. Compared to raw lactose (Figure 1a), the surface morphology of porous lactose (Figure 1c–e) changed significantly. First, the porous lactose exhibited a spheroidal shape. Second, the porous lactose showed a porous and fluffy structure. Third, the hollow pore structure appeared with increasing the amount of PVP K30 (Figure 1d,e). These changes are often conducive to improved dissolution behavior of health functional foods and drugs, which was discussed in detail in Section 3.3. As Lactose-0 was prepared without PVP K30 during spray drying, thus, the particles couldn’t exhibit a complete spherical structure, but a flake hemispherical shape. Possibly due to the impact of the solvent effect on the structure, some heterogeneous pores were observed in the Lactose-0.

### 3.2. Physical Characterization of Materials

#### 3.2.1. Powder Properties

The powder properties of materials were summarized in Table 1. Porous lactose was successfully prepared in this study. Furthermore, the higher porosity and surface area enabled a more homogeneous particle size distribution, better flowability, and more fluffy structure that were all conducive to the downstream operating process for the preparation of health functional foods/drugs [23,24].

Firstly, the values of SA-BET, SA-BJH, PV, and PD quantificationally confirmed that co-spray drying assisted with PVP K30 as templating agent, then ethanol washing PVP K30 did prepare porous lactose successfully. Compared to raw lactose, (i) the SA-BET of Lactose-P1, Lactose-P2, and Lactose-P3 increased 41.9%, 40.0%, and 82.8%, respectively; (ii) similarly, the SA-BJH of Lactose-P1, Lactose-P2 and Lactose-P3 increased 68.3%, 69.4%, and 134.2%, respectively; (iii) Lactose-P1, Lactose-P2, and Lactose-P3 exhibited 21.6-fold, 11.9-fold, and 13.0-fold higher PV, respectively; and (iv) Lactose-P1, Lactose-P2, and Lactose-P3 exhibited 12.4-fold, 6.6-fold, and 5.0-fold higher PD, respectively. Compared to raw lactose-0, the SA-BET and SA-BJH of Lactose-P1, Lactose-P2, and Lactose-P3 did not increase; however, (i) the PV of Lactose-P1, Lactose-P2 and Lactose-P3 increased 5.7-fold, 2.8-fold, and 3.2-fold, respectively; and (ii) the PD of Lactose-P1, Lactose-P2, and Lactose-P3 increased 10.6-fold, 5.5-fold, and 4.1-fold, respectively.

Secondly, it is interesting to find that the porous lactose showed smaller particle size and more homogeneous particle size distribution simultaneously when compared with raw lactose and Lactose-0. The smaller the values of span and uniformity are, the more uniform the particle size distribution of the material is. Commonly, the smaller the particle size, the more heterogeneous the particle size distribution [25,26]. However, compared to raw lactose, the d (0.5), span and uniformity values simultaneously decreased 28.7%, 17.7%, and 23.4% for Lactose-P1, 18.1%, 21.6%, and 29.1 for Lactose P2, 36.2%, 24.1%, and 33.7% for Lactose P3, respectively. When compared to Lactose-0, these values of porous lactose dropped more significantly. This might be attributed to the following aspects: (i) the particle size of material was mainly determined by the processing method; (ii) it has been reported that the spray-dried material consisted of porous, spherical agglomerates of solid particles that were fairly uniform in size because of the spherical nature of liquid particles after evaporation of water. Meanwhile, the particle size distribution of the spray- dried material was controlled by the atomization process and the type of drying chamber [27,28], and (iii) the addition of PVP K30 reduced the surface tension of the solution [29], thus, resulting in smaller and more uniform particles during spray drying.

Thirdly, it is also intriguing that porous lactose exhibited better flowability when compared with raw lactose and Lactose-0. AR, CI, and HR were often utilized as the indicators to characterize and describe the flowability of materials [29,30]. Generally, the smaller the values, the better the flowability of the material [25]. Compared to raw lactose, the AR, CI, and HR of porous lactose decreased 25.6–29.4%, 31.2–39%, and 21.7–25.7%, respectively. Similar results were found when they were compared with Lactose-0. The better flowability of porous lactose could be due to the spheroidal shape and homogeneous particle size distribution. Relatively spheroidal shape and uniform particle size are conducive to improving the flowability and decreasing the values of AR, CI, and HR [31,32,33]. Small particles often led to poor flowability, but porous lactose exhibited the opposite phenomenon. This illustrated that the particle shape and particle size distribution of materials showed stronger influence on the flowability than the particle size in a certain particle size range.

Fourthly, the porous lactose exhibited lower ρ_b_ and ρ_t_, indicating that they were fluffier, and exhibited better filling ability and health functional food/drug loading ability. This was due to the high porosity and surface area. Moreover, these are also conducive to the downstream operating process for the preparation of health functional foods/drugs, as it has been reported that (i) the high porosity could strengthen the mechanical interlocking between particle surfaces under pressure, thus resulting in strong compacts; and (ii) high surface area could provide strong adhesion sites for particles, hence resulting in less segregation within the powder mixtures [3,34,35].

#### 3.2.2. XRD

**Figure 2:**
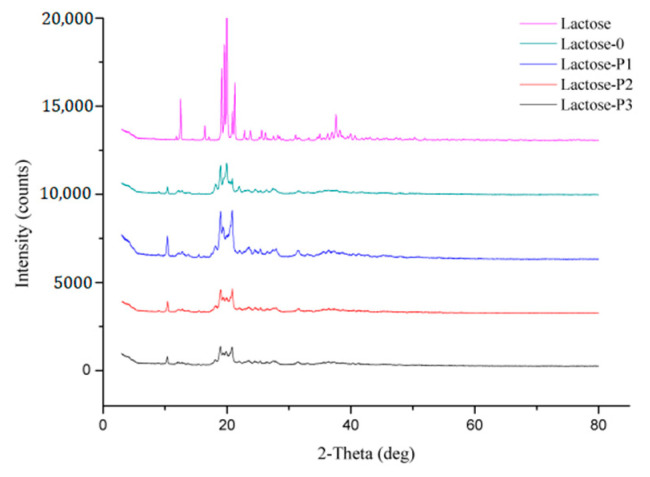
Representative X-ray diffractograms of materials. Lactose, the raw material; Lactose-0, processed without PVP K30; Lactose-P1, processed with 1% PVP K30; Lactose-P2, processed with 2% PVP K30; Lactose-P3, processed with 3% PVP K30.

The XRD results, summarized in Figure 2, demonstrated that the crystal form of lactose was changed during the spray drying.

First, the both the Lactose-0 and porous lactose changed crystal form during spray drying. It has been reported that spray drying process itself could lead to the formation of amorphous parts and change the crystallinity of obtained particles by rearrangement of the physical state [19,25,36,37]. In general, the specific diffraction angles (2θ) are 12.5°, 16.4°, and 20.0° for α-lactose monohydrate, respectively. Those for anhydrous β-lactose are 10.5° and 20.9°, respectively. Those for the mixture of α- and β-lactose are 19.1° and 20.0° (α:β molar ratio 5:3) and 19.5° (α:β molar ratio 4:1), respectively [38]. Therefore, it can be seen from Figure 2 that raw lactose is α-lactose monohydrate. While the 12.5 degree peak disappears, it is replaced by a peak of 10.5 degrees for all spray-dried samples, indicating that the crystal phase has changed for all spray-dried lactose, and both of them exhibit an anhydrous β-lactose structure.

Second, the porous lactose also exhibited a different crystal form when compared with Lactose-0. Specifically embodied in the peak with an angle of 2θ = 19.96 disappears. The crystal form of materials is mainly affected by the crystal habit, which can be modified by the presence of small amounts of a crystal modifier in the crystallization medium. Some hydrophilic polymers, such as PVP and hydroxypropyl cellulose, are commonly used for such a purpose [25,32]. Therefore, we speculate that in this study the hydrophilic polymer PVP K30 acts as a crystal modifier during the spray-drying process, so that porous lactose and Lactose-0 have different crystal types.

Third, it is well known that lactose exists in two isomeric forms, α-lactose and β-lactose, and can be either crystalline or amorphous [28]. However, compared to a crystal, the amorphous material is generally unstable and has a tendency tend to form crystalline material.

In summary, the spray-dried lactose (Lactose-0, Lactose-P1, Lactose-P2, and Lactose-P3) was mainly in crystalline structure and changed the crystal form, which was consist with Figure 2.

#### 3.2.3. FTIR

**Figure 3:**
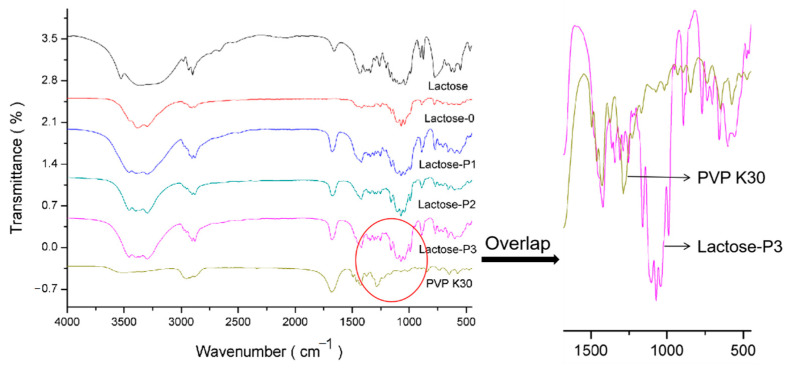
The FTIR of materials. Lactose, the raw material; Lactose-0, processed without PVP K30; Lactose-P1, processed with 1% PVP K30; Lactose-P2, processed with 2% PVP K30; Lactose-P3, processed with 3% PVP K30.

The FTIR spectrogram for materials were compared in Figure 3. Similar with the results of XRD, the results of FTIR confirmed that (i) Lactose-P1, Lactose-P2, and Lactose-P3 showed similar infrared spectrogram, (ii) the raw lactose, Lactose-0, and porous lactose exhibited different infrared spectrograms. As can be observed from Figure 3, different characteristic peaks among raw lactose, Lactose-0, and porous lactose were found around 3500–2800 cm^−1^ and 1750–500 cm^−1^. The raw lactose showed an apparent absorption peak at 3520 cm^−1^, which was due to the free O-H vibrations from the water molecules, and was the characteristic band for pure α-lactose monohydrate. Moreover, it seems that the three peaks at 920 cm^−1^, 900 cm^−1^, and 875 cm^−1^ were merged together to give a broad peak at 892 cm^−1^. The high value for the peak areas at these characteristic peak positions indicates that raw lactose was amorphous lactose. This observation was in good agreement with understanding that (i) spray-dried lactose changed crystal form, and (ii) the porous lactose exhibited different crystal form with Lactose-0. FTIR spectrogram of PVP K30 measured under the same conditions was added in the FT-IR picture. It could be observed that PVP K30 exhibited an obvious absorption peak near 1278 cm^−1^, which belongs to O-H in-plane bending vibration. while all other lactose samples showed no absorption peak at all. It is also very interesting that all samples have a peak near at 1654 cm^−1^, which respectively represents the bending vibration of the hydroxyl groups of the crystalline water for Lactose (1650 cm^−1^) [39], and C = C stretching vibration in alkene bonds for PVP K30 [40], besides Lactose-0. That’s because Lactose-0 loses the crystalline water after the spray-drying process.

#### 3.2.4. TG and DTG

**Figure 4:**
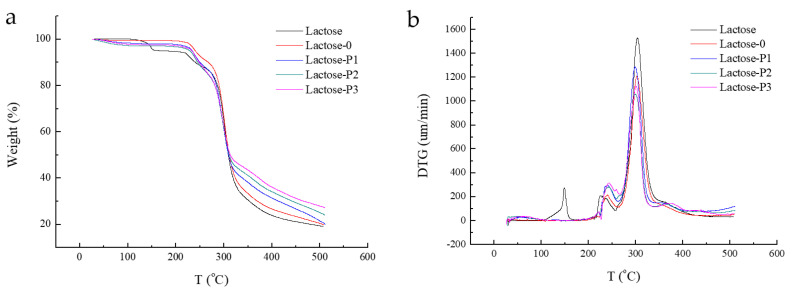
The (**a**) TG and (**b**) DTG analysis of materials. Lactose, the raw material; Lactose-0, processed without PVP K30; Lactose-P1, processed with 1% PVP K30; Lactose-P2, processed with 2% PVP K30; Lactose-P3, processed with 3% PVP K30.

The thermal stability of materials was investigated by TGA. As observed from Figure 4, the raw lactose exhibited a pattern of three-step weight loss in the temperature range of 0–500 °C. The first sharp weight loss was in the range of 100–150 °C, which corresponded to the loss of the water bound in the crystalline lattice; then a slower step degrade weight loss appeared between 210 °C and 240 °C; and a faster weight loss appeared until 255 °C and obtained a mass balance at 310 °C, corresponding to the crystal water in α-lactose monohydrate. However, the porous lactose and Lactose-0 showed a pattern of two-step weight loss in the same temperature range. A slight weight loss first appeared in the range of 225–245 °C; then a fast weight loss appeared at 265 °C and obtained a mass balance at 300 °C, which corresponded to the melt peaks of β-lactose. Moreover, the weight loss of porous lactose, as a whole, was less than Lactose-0, which was probably due to the form of crystals in porous lactose. The crystalline state has better thermal stability than the amorphous state. That was to say, porous lactose showed good thermal stability.

### 3.3. In Vitro Dissolution Behavior

**Figure 5:**
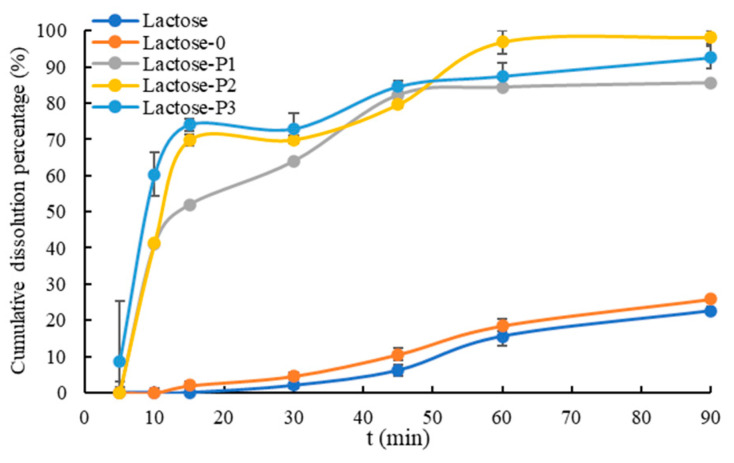
In vitro dissolution behavior profiles of Cur-loaded powder. Lactose, the raw material; Lactose-0, processed without PVP K30; Lactose-P1, processed with 1% PVP K30; Lactose-P2, processed with 2% PVP K30; Lactose-P3, processed with 3% PVP K30.

The in vitro dissolution behavior of capsules containing curcumin-loaded lactose was evaluated in Figure 5. The curcumin-loaded Lactose-0 exhibited a comparable dissolution behavior with raw lactose. Both them showed a very slow dissolution speed. Moreover, the cumulative dissolution percentage of capsules containing curcumin-loaded raw lactose and Lactose-0 at 60 min were 15% and 19%, respectively. At 90 min, the cumulative dissolution percentage of capsules containing curcumin-loaded raw lactose and Lactose-0 were still only 23% and 26%, respectively.

Compared to raw lactose and Lactose-0, the capsules containing curcumin-loaded porous lactose exhibited a significantly improved dissolution behavior. All of them showed a very fast dissolution speed from 5 min to 15 min. The capsules containing curcumin-loaded Lactose-P1 and Lactose P3 had almost reached the dissolution equilibrium at 45 min, and capsule containing curcumin-loaded Lactose-P2 also reached the dissolution equilibrium at 60 min. The cumulative dissolution percentage of capsules containing curcumin-loaded Lactose-P1, Lactose-P2, and Lactose-P3 at 90 min were 86%, 98%, and 93%, respectively.

The similarity factor (f_2_) (Table 2) was also calculated based on the first six points to compare the effect of different lactose on the dissolution behavior of curcumin. When the f_2_ < 50 is taken as the criterion for inequivalence, and the 50 ≤ f_2_ ≤ 100 is meaning equivalence for the dissolution behavior [41]. The f_2_ also demonstrated that (i) it was equivalent for the dissolution behavior of curcumin-loaded raw lactose and Lactose-0; (ii) it was inequivalent for the dissolution behavior of curcumin-loaded porous lactose and raw lactose/Lactose-0; (iii) it was equivalent for the dissolution behavior of curcumin-loaded Lactose-P2 and Lactose-P1/-P3; and (iv) the f_2_ between Lactose-P1 and -P3 was slightly less than 50, meaning it, to a certain degree, was inequivalent for the dissolution behavior of curcumin-loaded Lactose-P1 and -P3.

Generally, the dissolution rate of a solid in solvent is positively related to SA [42], and is written by the Noyes–Whitney equation, as follows:

(4) $$\frac{dC}{dt}=\frac{DS_{w}}{Vh}\left(C_{S}-C\right)$$

where *C* and *C_s_* represent the concentration of the dissolved substance at a given time t and the solubility concentration of the substance, respectively. *D*, *S_w_*, *V*, and *h* represent the diffusion coefficient of the substance, the surface area of exposed solid, the volume of solution, and the thickness of the diffusion layer, respectively [43].

Therefore, it was reasonable that the capsules containing curcumin-loaded porous lactose exhibited significant faster dissolution behavior than raw lactose and Lactose-0 because of the higher SA, PV, and PD, which was discussed in detail in Section 3.2.1.

However, intriguingly, an opposite result was also observed in the dissolution behavior of capsules containing curcumin-loaded raw lactose and Lactose-0. That is, although Lactose-0 showed three times higher SA and PV than raw lactose, curcumin-loaded Lactose-0 and raw lactose exhibited similar dissolution behavior. This might be attributed to the small PD and inhomogenous particle size distribution of Lactose-0. In fact, the PD of raw lactose and Lactose-0 were similar. Meanwhile, the high SA and PV were because of the hollow or porous structure due to the inhomogenous particle size distribution, which contains the smallest-sized particles.

Furthermore, the dissolution behavior of capsules containing curcumin-loaded porous lactose was also compared. As a whole, the dissolution behavior of capsule containing curcumin-loaded porous lactose was improved with increasing the use level of PVP K30 and surface area. Although Lactose-P1 had the biggest PV and PD among the porous lactose, the capsule containing curcumin-loaded Lactose-P1 showed the lowest dissolution behavior among the three. With increasing the use level of PVP K30, the porous structures of lactose increased, and some hollow structures of lactose also appeared, e.g., the red arrow in Figure 1. As the hollow structures were not considered pore structures, they were not counted in PV and PD; they can, however, be counted in SA, and contributed to the improved dissolution behavior.

## 4. Conclusions

As is known to all, lactose is the most common excipient in health functional food and pharmaceutical industry, and it is needed to prepare porous lactose in order to increase the loading capacity and improve the dissolution behavior of insoluble active ingredients. Nowadays, the co-spray drying assisted with templating agent is the most popular method for the production of porous material.

In this study, PVP K30 was firstly utilized as templating agent with different use level to prepare porous lactose. Then, the surface morphology, surface area, pore volumes, pore diameter, powder properties, X-ray diffraction, Fourier transform infrared spectrometer, thermal gravity, and differential thermal gravity were profoundly characterized. Finally, the porous lactose was also employed as a health functional food carrier to explore the effect on the dissolution behavior of curcumin. The results demonstrated that (i) porous lactose was successfully prepared using PVP K30 as templating agent; (ii) PVP K30 significantly improved the yield of lactose in the spray-drying; (iii) the improved powder properties of porous lactose were more conducive to the downstream operating process for the preparation of health functional foods/drugs; and (iv) the porous lactose significantly improved the dissolution behavior of curcumin.

The results obtained could provide new perspectives, theoretical support, and guidance on the development of porous materials. Therefore, this study might be significant in facilitating the development of health functional food.

## 5. Patents

There is a patent (ZL202011585742.7) resulting from the work reported in this manuscript.

## Figures and Tables

**Table 1:** The characterization of the materials studied in this work.

Materials	Yield (%)	ρb (g/mL)	ρt (g/mL)	CI	HR	AR (°)	d (0.5) (μm)	Span	Uniformity	SA-BET (m^2^/g)	SA-BJH (m^2^/g)	PV (cm^3^/g)	PD (nm)
Lactose		0.458 ± 0.007	0.864 ± 0.004	47.0 ± 1.0	1.89 ± 0.04	56.7 ± 0.7	42.5 ± 0.5	2.69 ± 0.07	0.927 ± 0.202	1.11 ± 0.02	1.25	0.00133	42.5
Lactose-0	69.0	0.329 ± 0.003	0.558 ± 0.005	41.0 ± 0.0	1.70 ± 0.00	45.3 ± 1.7	45.9 ± 0.2	3.40 ± 0.03	1.010 ± 0.011	3.44 ± 0.01	3.62	0.00447	49.4
Lactose-P1	85.3	0.398 ± 0.008	0.583 ± 0.007	31.7 ± 1.5	1.46 ± 0.03	42.2 ± 1.4	30.3 ± 0.1	2.21 ± 0.02	0.710 ± 0.012	1.58 ± 0.28	2.11	0.03010	570.0
Lactose-P2	85.5	0.397 ± 0.003	0.557 ± 0.011	28.7 ± 1.2	1.40 ± 0.02	40.5 ± 0.8	34.8 ± 0.1	2.11 ± 0.01	0.657 ± 0.007	1.56 ± 0.43	2.12	0.01720	323.0
Lactose-P3	89.1	0.376 ± 0.004	0.555 ± 0.007	32.3 ± 0.6	1.48 ± 0.01	40.0 ± 1.3	27.1 ± 0.1	2.04 ± 0.00	0.615 ± 0.002	2.03 ± 0.29	2.94	0.01870	254.0

Lactose, the raw material; P, polyvinylpyrrolidone (PVP K30); Lactose-0, processed without PVP K30; Lactose-P1, processed with 1% PVP K30; Lactose-P2, processed with 2% PVP K30; Lactose-P3, processed with 3% PVP K30; ρ_b_, bulk density; ρ_t_, tapped density; CI, Carr’s index; HR, Hausner ratio; AR, angle of repose; d (0.5), median particle size; span, particle size distribution; SA-BET, the surface area characterized by BET; SA-BJH, the surface area characterized by BJH; PV, BJH. Adsorption cumulative volume of pores between 17.000 nm and 3000.000 nm diameter; PD, BJH Adsorption average pore diameter.

**Table 2:** The f_2_ of capsules containing curcumin-loaded lactose studied in this work.

f_2_	Lactose	Lactose-0	Lactose-P1	Lactose-P2	Lactose-P3
Lactose	100.0				
Lactose-0	77.9	100.0			
Lactose-P1	12.6	13.6	100.0		
Lactose-P2	10.3	11.2	51.5	100.0	
Lactose-P3	9.3	10.0	44.1	50.3	100.0

## Data Availability

The raw/processed data required to reproduce these findings cannot be shared at this time due to technical or time limitations. Data will be made available on request.
